# Molecular Self-Assembly and Adsorption Structure of 2,2′-Dipyrimidyl Disulfides on Au(111) Surfaces

**DOI:** 10.3390/molecules29040846

**Published:** 2024-02-14

**Authors:** Dongjin Seo, Sicheon Seong, Haeri Kim, Hyun Su Oh, Jun Hyeong Lee, Hongki Kim, Yeon O Kim, Shoichi Maeda, Shunta Chikami, Tomohiro Hayashi, Jaegeun Noh

**Affiliations:** 1 Department of Chemistry, Hanyang University, Seoul 04763, Republic of Korea; nammi72795@hanyang.ac.kr (D.S.); ssc09122@hanyang.ac.kr (S.S.); kkhman2548@hanmail.net (H.K.); ohs01145@naver.com (H.S.O.); mystlee16@hanyang.ac.kr (J.H.L.); khg2202@naver.com (H.K.); audwls8925@naver.com (Y.O.K.); 2 Department of Materials Science and Engineering, School of Materials and Chemical Technology, Tokyo Institute of Technology, Yokohama 226-8503, Japan; maeda.s.am@m.titech.ac.jp (S.M.); chikami.s.aa@m.titech.ac.jp (S.C.); 3 Research Institute for Convergence of Basic Science, Hanyang University, Seoul 04763, Republic of Korea

**Keywords:** 2,2′-dipyrimidyl disulfide, 2-pyrimidinethiol, self-assembled monolayers, adsorption, surface structure, scanning tunneling microscopy, X-ray photoelectron microscopy

## Abstract

The effects of solution concentration and pH on the formation and surface structure of 2-pyrimidinethiolate (2PymS) self-assembled monolayers (SAMs) on Au(111) via the adsorption of 2,2′-dipyrimidyl disulfide (DPymDS) were examined using scanning tunneling microscopy (STM) and X-ray photoelectron spectroscopy (XPS). STM observations revealed that the formation and structural order of 2PymS SAMs were markedly influenced by the solution concentration and pH. 2PymS SAMs formed in a 0.01 mM ethanol solution were mainly composed of a more uniform and ordered phase compared with those formed in 0.001 mM or 1 mM solutions. SAMs formed in a 0.01 mM solution at pH 2 were composed of a fully disordered phase with many irregular and bright aggregates, whereas SAMs formed at pH 7 had small ordered domains and many bright islands. As the solution pH increased from pH 7 to pH 12, the surface morphology of 2PymS SAMs remarkably changed from small ordered domains to large ordered domains, which can be described as a (4√2 × 3)R51° packing structure. XPS measurements clearly showed that the adsorption of DPymDS on Au(111) resulted in the formation of 2PymS (thiolate) SAMs via the cleavage of the disulfide (S-S) bond in DPymDS, and most N atoms in the pyrimidine rings existed in the deprotonated form. The results herein will provide a new insight into the molecular self-assembly behaviors and adsorption structures of DPymDS molecules on Au(111) depending on solution concentration and pH.

## 1. Introduction

Self-assembled monolayers (SAMs) provide a very convenient and powerful method to control the surface and interface characteristics of various metal surfaces via modifying the chemical structure of adsorbates with different active anchoring groups and molecular backbones [1,2,3,4,5,6,7,8,9,10]. Due to their advantages, SAMs have been applied to various nanotechnological applications in nanopatterning [1], biosensors [11], bioelectronics [12], solar cells [13,14], and molecular electronics [15,16,17,18]. In particular, aromatic thiol SAMs with π-conjugated molecular backbones have attracted a considerable interest due to their interesting electrical properties and controllable charge transfer behaviors in electronic devices [19,20,21,22,23]. The physical properties of SAM-modified electronic devices are significantly influenced by the surface structures of SAMs such as molecular orientation, structural order, surface coverage, and structural defects of adlayers [24,25,26].

Interestingly, the SAMs derived from heteroaromatic thiols containing nitrogen atom(s) on gold surfaces have also drawn much attention because they can provide unique interfacial properties for understanding the electron transfer reactions of various metalloproteins, such as cytochrome c, myoglobin, and ferredoxin [27,28,29]. It was revealed that the charge transfer behaviors of cytochrome c were strongly influenced by the position of nitrogen in the aromatic ring and the adsorption geometry of SAMs [27,29]. The SAMs of 4-pyridinethiol (4PySH) effectively promoted an electron transfer reaction between the gold electrode and cytochrome c, while the SAMs of 2-pyridinethiol (2PySH) were much less effective [27,29]. This is because the nitrogen atom attached at the 2-position of 2PySH can interact more easily than the nitrogen atom attached at the 4-position of 4PySH with gold surfaces during SAM formation, as revealed by scanning tunneling microscopy (STM) [29]. Therefore, the nitrogen atoms in 4PyS SAMs can act as an important interfacial layer to transfer electrons between the gold electrode and cytochrome c. The SAMs of 2-pyrimidinethiol (2PymSH) also showed a facile electron transfer reaction between the gold surface and cytochrome c, as in the case of 4PyS SAMs [27]. Several spectroscopic studies were performed to examine the adsorption processes and geometry of these N-heteroaromatic thiolate SAMs [30,31,32]. These SAMs were mainly formed via the chemisorption of thiol groups onto gold surfaces, while the nitrogen atom(s) of an aromatic ring can also interact with the gold surfaces, so that the surface structures of these heteroaromatic thiolate SAMs were different from those of normal aromatic thiolate SAMs derived from benzenethiols [33,34,35]. STM observations also revealed that the formation and molecular arrangements of 2PyS, 4PyS, and 2PymS SAMs were different from each other and depended on the position and number of nitrogen atoms in the aromatic ring [29,31,36]. 2PymSH has often been used as a suitable matrix for the fabrication of functional supramolecular assemblies with a great diversity of two-dimensional (2D) molecular layers [37]. The first STM images for 2PymS SAMs on Au(111) showed a very unique surface structure with a “ladder-like molecular orientation” [36]. However, it is difficult to analyze the molecular packing structure of these SAMs due to poor STM resolution.

**Figure 1:**
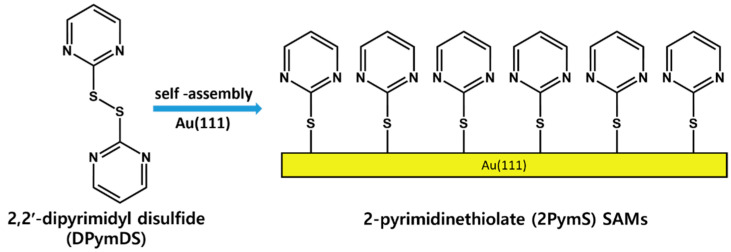
The chemical structure of DPymDS and the formation of 2PymS SAMs on Au(111).

It is important to understand the formation and surface features of 2PymS SAMs on Au(111) at a molecular scale, from both a fundamental and practical point of view [1,2,3]. Therefore, 2PymS SAMs on Au(111) derived from the 2,2′-dipyrimidyl disulfide (DPymDS) were examined using STM and X-ray photoelectron spectroscopy (XPS) as a function of solution concentration and pH. Figure 1 shows the chemical structure of DPymDS and a schematic view for the formation of 2PymS SAMs on Au(111) from DPymDS. It is well known that 2PymSH shows thione–thiol tautomerization in solution or gas phase [32,38], and it is expected that these complicated structural transitions in solution hinder the formation of 2D ordered SAMs. To avoid this problem, DPymDS was synthesized as a target molecule for the formation of ordered 2PymS SAMs on Au(111) in this study. Herein, we report the first high-resolution STM results showing well-ordered 2PymS SAMs on Au(111) formed in a 0.01 mM ethanol solution at pH 12, which can be described as a (4√2 × 3)R51° structure.

## 2. Results and Discussion

### 2.1. Concentration Effect on the Formation of 2PymS SAMs on Au(111) from DPymDS

It has been demonstrated that the adsorption of organic disulfides or organic thiols on gold surfaces occurs via the cleavage of the S-S bonds of organic disulfide or the cleavage of the S-H bond in organic thiols, resulting in the formation of identical gold-bound thiolate SAMs [39,40,41]. Therefore, it was reasonable to consider that the adsorption of DPymDS on Au(111) results in the formation of 2PymS SAMs, as shown in the schematic view in Figure 1. The formation of these thiolate SAMs was demonstrated through our XPS study, which will be discussed later. The solution concentration of SAM preparation is an important parameter for determining the growth processes and surface structures of organic thiolate SAMs [1,42,43,44]. As an example, the adsorption of OH-terminated undecanethiols on Au(111) in a 0.01 mM aqueous solution led to the formation of a densely packed standing-up phase via a striped phase, in which the molecular backbones are oriented parallel to the surface, while they would not form a striped phase in a 0.05 mM solution due to a fast adsorption process in a high solution concentration [43]. Moreover, the domain formation and structural order of pentachlorobenzenethiolate (PCBT) SAMs on Au(111) were considerably influenced by solution concentration. Long-range ordered PCBT SAMs were observed in a 0.1 mM ethanolic solution, whereas short-range ordered monolayers with many structural defects were formed in a 1 mM solution [44]. Therefore, the effect of solution concentration is very important to understand the formation and structure of 2PymS SAMs on Au(111).

**Figure 2:**
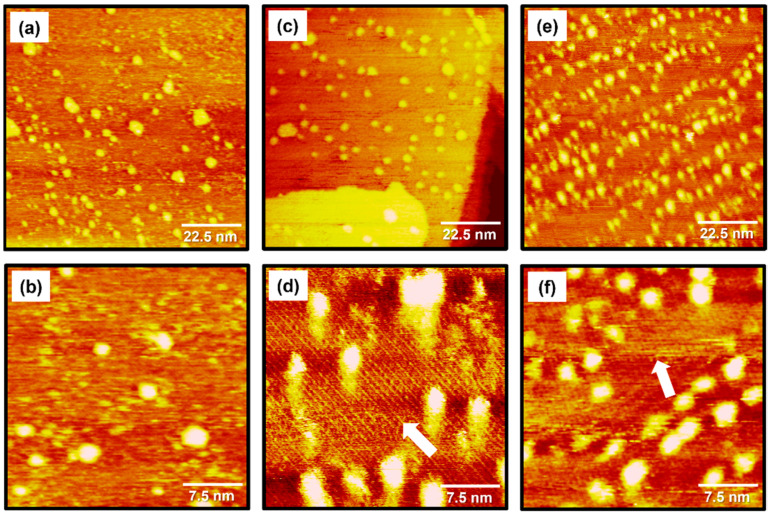
STM images of 2PymS SAMs on Au(111) in a 1 mM ethanol solution of DPymDS for 1 h as a function of solution concentration: (**a**,**b**) 0.001 mM, (**c**,**d**) 0.01 mM, and (**e**,**f**) 1 mM. Scan sizes of STM images were (**a**,**c**,**e**) 90 nm × 90 nm and (**b**,**d**,**f**) 30 nm × 30 nm. An ordered phase containing rows of molecules are marked by white arrows.

STM images in Figure 2 show the structural morphologies of 2PymS SAMs on Au(111) formed in an ethanolic solution as a function of solution concentration: (Figure 2a,b) 0.001, (Figure 2c,d) 0.01, and (Figure 2e,f) 1 mM. STM observations revealed that the formation and structural order of 2PymS SAMs were markedly influenced by solution concentration. The SAMs formed at the low concentration of 0.001 mM were mainly made of a disordered phase with many bright islands across the gold surface, as shown in Figure 2a,b. Interestingly, an ordered phase containing rows of molecules marked by white arrows and bright islands for 2PymS SAMs were observed when the solution concentration increased to 0.01 mM (Figure 2c,d). The magnified STM image (30 nm × 30 nm) in Figure 2d shows ordered molecular rows with an intermolecular distance of 16.5 ± 0.3 Å. On the other hand, when the solution concentration increased to 1 mM, the number of bright islands significantly increased compared with those formed in 0.001 or 0.01 mM solutions (Figure 2a,c,e). The magnified STM image (30 nm × 30 nm) in Figure 2f also shows the formation of ordered phases for 2PymS SAMs on Au(111) containing rows of molecules marked by a white arrow. We speculate that the ordered phase was composed of the paired molecular rows. Structural details will be discussed later. However, the observed bright islands significantly interfered with the high-resolution STM images for the ordered phase of SAMs, as shown in Figure 2f. Therefore, it was expected that the SAM samples prepared in a 0.01 mM solution would be more suitable to obtain molecular-scale information for the surface structures of 2PymS SAMs. In addition, similar bright islands were usually observed for aromatic thiolate SAMs derived from benzenethiols or substituted benzenethiol derivatives [16,34,35,45]. The formation mechanisms for these islands on gold surfaces were well described in the literature [34]. The bright islands with protrusions of approximately 2.5 Å from the surface could be Au adatom islands covered with SAMs, which resulted from the low mobility of SAM-covered Au adatom islands during SAM formation [34]. The height of bright islands for 2PymS SAMs varied from 0.1 to 2.5 Å. This result means that the bright islands were composed of two different components: (1) SAM covered with Au adatom islands with a protrusion of approximately 2.5 Å and (2) molecular aggregates with a protrusion of less than about 2.0 Å. Spectroscopic measurements suggested that molecular aggregates could be more easily formed via the hydrogen bonding interactions between 2PymSH molecules compared with benzenethiol derivatives [32,45], as shown in this STM observation.

### 2.2. The Formation of 2PymS SAMs on Au(111) from DPymDS at Different pHs

It was reported that SAMs on metal surfaces formed by heteroaromatic thiols containing nitrogen atoms, such as pyridinethiol or pyrimidinethiol derivatives, showed pH-dependent structural changes due to the protonation and deprotonation of nitrogen atoms in the heterocyclic ring [30,45,46]. Surface-enhanced Raman scattering measurements suggested that the adsorption geometry of 2PyS SAMs on silver nanoparticles formed at pH values lower than the pK_2_ value changed from the tilted orientation to the flat orientation of adsorbed molecules with respect to the surface due to N-protonation, while that of 2PymS SAMs formed a flat orientation regardless of N-protonation [30]. STM observations revealed the pH-dependent phase transition of 4PyS SAMs on Au(111) from a *p*(10 × √3)R30° packing structure (in acidic solution) to a *p*(10 × √3)R30° packing structure (basic solution) [45].

**Figure 3:**
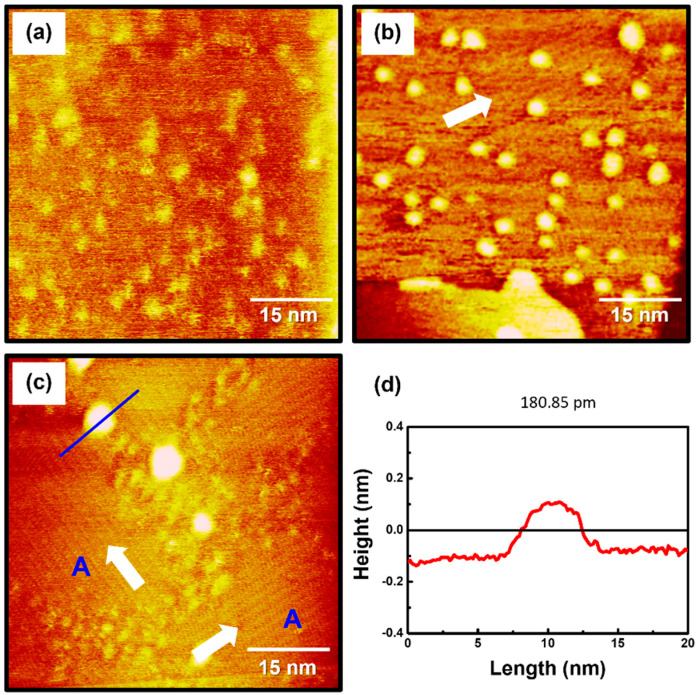
STM images of 2PymS SAMs on Au(111) in a 0.1 mM ethanol solution of DPymDS for 1 h as a function of solution pH: (**a**) pH = 2, (**b**) pH = 7, and (**c**) pH = 12. Scan size of all STM images was 60 nm × 60 nm. Domain angles between two ordered domains are indicated by the white arrows. Large ordered domains are marked (region A), (**d**) Height profile along the blue line on the bright aggregate in STM image (**c**).

To examine the formation and surface features of 2PymS SAMs on Au(111), the SAMs were prepared in an optimized solution concentration of 0.01 mM at three pH conditions: pH 2, pH 7, and pH 12. STM observations revealed that the formation and structure of the SAMs were significantly influenced by solution pH, as shown in Figure 3. The STM image in Figure 3a shows that the SAMs at pH 2 were composed of a fully disordered phase with many irregular and bright aggregates that were not clearly visualized. This means that the crystallized and ordered phases have difficulty forming under acidic conditions. Charge repulsion between N-protonated 2PymS molecules upon adsorption on Au(111) significantly prevents the formation of the ordered phase, as demonstrated by this STM study. On the other hand, the SAMs formed at pH 7 (neutral) contained ordered molecular rows and many bright islands with a round shape, as shown in Figure 3b. The size of ordered domains is less than approximately 30 nm, and the size of bright islands is in the range of 2–4 nm. The ordered molecular rows were clearly visualized in the magnified STM image (30 nm × 30 nm) of Figure 2d. However, it was difficult to obtain molecular-scale STM images of ordered domains because the many bright islands protruding from the SAM surface induced an instability of tunneling currents during the scanning of STM tips over SAM samples. As the solution pH increased from pH 7 to pH 12, the surface structures of 2PymS SAMs remarkably changed from small ordered domains and many bright islands to large ordered domains (region A) and a few bright islands (Figure 3c). The sizes of ordered domains became larger than approximately 30 nm, and the size of bright islands also increased in the range of 3–6 nm. Domain angles between two ordered domains, indicated by the white arrows, were measured to be 93 ± 2°, which means that the observed domain orientations deviated from the hexagonal symmetry of the Au(111) lattice, suggesting that the formation and growth of 2PymS SAMs on Au(111) are influenced by the van der Waals interactions between pyrimidine molecular backbones. Figure 3d shows a height profile along the blue line above the bright island in Figure 3c showing a protrusion of approximately 2.5 Å from the surface. This is good evidence that the bright islands are the SAM-covered adatom islands, as discussed above. From our STM image, we demonstrated that long-range ordered SAMs on Au(111) could be formed under a basic solution when two nitrogen atoms of the pyrimidine ring in DPymDS molecules are deprotonated during SAM formation.

### 2.3. Surface Structure of Well-Ordered 2PymS SAMs on Au(111)

**Figure 4:**
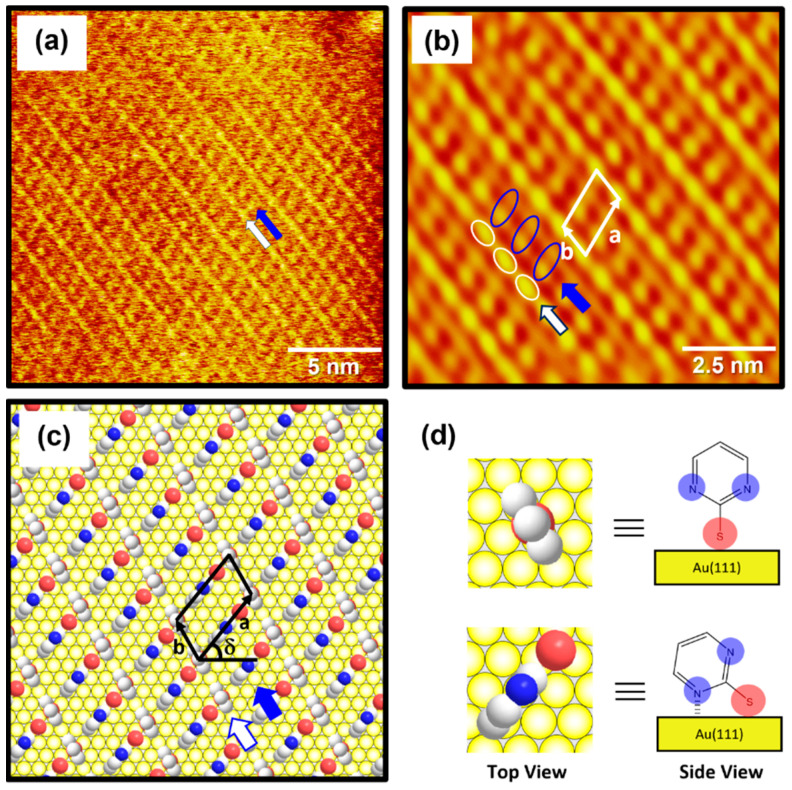
(**a**) STM image (20 nm × 20 nm) of highly ordered 2PymS SAMs on Au(111) formed in the solution at pH = 12. (**b**) Low-pass filtered STM image (10 nm × 10 nm) showing high periodicities in the molecular arrangements of 2PymS SAMs. (**c**) A proposed structural model of the SAMs. (**d**) Top and side views of 2PymS molecules in the structural model of subfigure (**c**). The ordered domains composed of alternating paired molecular rows are indicated by white and blue arrows. Note that the white, red, and blue colors correspond to the carbon, sulfur, and nitrogen atoms in the structural model.

The high-resolution STM image (20 nm × 20 nm) in Figure 4a shows the molecular arrangement of 2PymS SAMs on Au(111) obtained from region A of Figure 3c, implying that the adsorption of DPymDS molecules on Au(111) at pH 12 led to the formation of highly ordered monolayers. The ordered domains were composed of alternating paired molecular rows indicated by white and blue arrows in Figure 4a. The low-pass filtered and magnified STM image (10 nm × 10 nm) of SAMs in Figure 4b clearly shows an oblique unit cell and the paired molecular rows containing one row with oval, bright molecular spots (the white open circles) and the other row showing bright and dark gradient molecular spots (the blue open circles). Based on the molecularly resolved STM image, the lattice parameters corresponding to the oblique unit cell containing two adsorbed molecules were extracted: a = 16.3 ± 0.2 Å = 4√2a_h,_ b = 8.8 ± 0.2 Å = 3a_h,_ and δ = 51°. Note that a_h_ is the interatomic distance of gold atoms, 2.89 Å. This ordered phase can be described as a (4√2 × 3)R51° structure. Figure 4c shows the proposed structural model of this ordered phase for 2PymS SAMs on Au(111) and Figure 4d shows the top and side views of pyrimidinethiolate molecules adsorbed on Au(111). It has been suggested that 2PymS molecules, which represent the brightest molecular spots in STM imaging, have an upright adsorption geometry, whereas the molecules that represent the dark and light gradient molecular spots have a tilted adsorption geometry, where one nitrogen atom in the pyrimidine ring interacts with the Au(111) surface. It is reasonable to consider that the brightest molecular spots in STM imaging contrast were caused by an upright adsorption structure of 2PymS molecules because they protruded more from the Au(111) surface compared to those with a tilted adsorption structure. It was also reported that the SAMs formed by N-heteroaromatic thiols such as 2-mercaptobenzimidazole and 2-mercaptobenzothiazole prefer a tilted adsorption structure rather than an upright structure, which is caused by the interactions between a heteroatom (N or S) in the aromatic ring and Au surface [47].

It was also found that the formation and packing structure of ordered domains for 2PymS SAMs with a (4√2 × 3)R51° structure were remarkably different from those reported in a previous paper [36], showing a mixed domain containing “ladder-like ordered rows” and “randomly oriented molecular rows”. However, the molecular-scale packing structure of this monolayer was not observed due to poor STM resolution. Such large differences in surface structures between our study and previous studies may come from the chemical structure of target molecules [DPymDS (disulfide) vs. 2PymSH (thiol)] used for SAM preparation in the experiment and the experimental conditions. It was reported that 2PymSH molecules can exist as the thiol or thione form due to tautomerization in solution, and the thione form is dominant in aqueous or ethanol solution [30,48], resulting in the formation of SAMs through hydrogen bonding networks [36]. However, DPymDS (disulfide) is hard to tautomerize in an ethanol solution and strong base (pH 12) due to the disulfide group of DPymDS. Hence, 2PymS SAMs on Au(111) formed from DPymDS have different domain and packing structures compared to those formed from 2PymSH. The average areal density (area per molecule) of 2PymS SAMs on Au(111) was calculated to be 66.83 Å^2^/molecule. On the other hand, STM observations revealed that the adsorption of 2-pyridinethiol with a nitrogen atom in a heteroaromatic ring on Au(111) led to the formation of ordered SAMs with a different (4 × √7)R40.9° packing structure [29]. Moreover, SAMs on Au(111) derived from benzenethiol (BT) without a nitrogen atom in the aromatic ring have different ordered domains with a (2 × 3√2)R23° packing structure, and the average areal density of BT SAMs on Au(111) was calculated to be 34.81 Å^2^/molecule [35], which is 1.9 times that of the closely packed monolayers compared with that for 2PymS SAMs. This result means that the nitrogen atom in the aromatic ring plays a dominant role in the domain formation and surface structures of SAMs.

### 2.4. S 2p XPS and N 1s Peaks for 2PymS SAMs on Au(111) at Different Solution pHs

**Figure 5:**
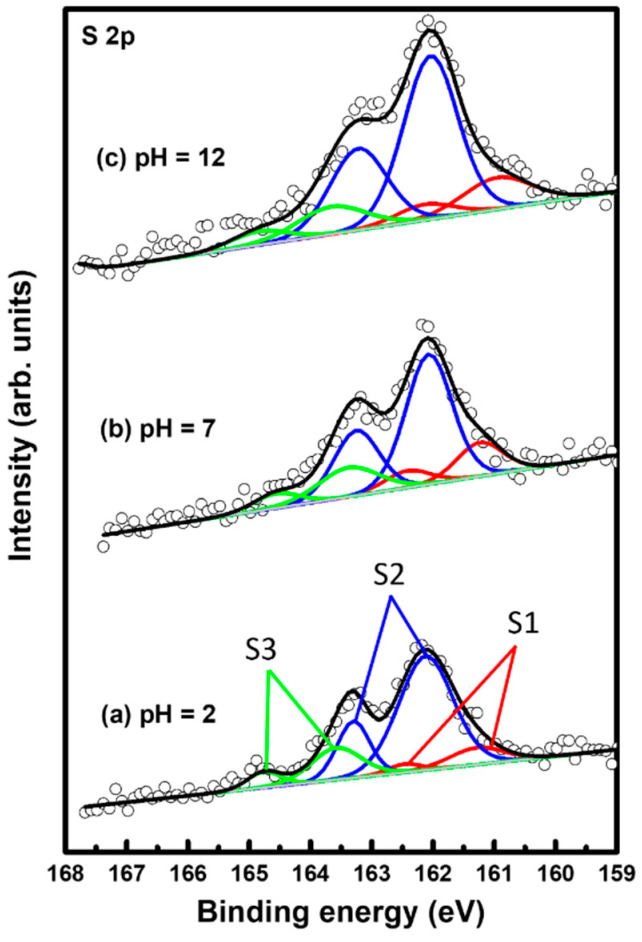
XPS spectra in the region of S 2p of 2PymS SAMs on Au(111) depending on solution pH: (**a**) pH = 2, (**b**) pH = 7, and (**c**) pH = 12. The three sulfur are peaks labeled S1 (red color), S2 (blue color), and S3 (green color).

The binding conditions of sulfur in 2PymS SAMs on Au(111) formed at different pH conditions were examined using XPS to understand the formation of SAMs from DPymDS. Figure 5 shows XPS peaks in the region of S 2p for 2PymS SAMs on Au(111) formed in a 0.01 mM at different solution pH values: (a) pH = 2, (b) pH = 7, and (c) pH = 12. It is well known that the S 2p peak appeared as a doublet consisting of 2p_3/2_ and 2p_1/2_ components in a 2:1 intensity ratio due to spin–orbital splitting [6,9,16,49,50,51]. XPS measurements revealed the existence of three sulfur peaks labeled S1 (red color), S2 (blue color), and S3 (green color) for 2PymS SAMs on Au(111). The binding energies of the 2p_3/2_ peaks of S1, S2, and S3 species for three SAM samples were observed at around 160.95–161.29, 162.04–162.12, and 163.38–163.68 eV, respectively, suggesting the presence of three sulfur species in the SAMs. S1 and S2 peaks are assigned to bound sulfurs on the gold surface, whereas the S3 peak is assigned to unbound sulfur, as demonstrated by many previous works [6,9,16,49,50,51,52,53]. Similarly bound S2 peaks with a strong intensity are usually observed for closely packed various thiolate SAMs on the gold surface, whereas similarly bound S1 peaks with weak intensity are often observed for loosely packed thiolate SAMs, and/or as a result of the existence of atomic sulfurs formed via the cleavage of S-C bonds during SAM formation of S-containing organic molecules, or formed via the thermal decomposition of thiolate SAMs on gold [6,49,50]. On the other hand, similarly unbound S3 peaks with weak intensity are usually observed in the range of 163–164 eV [49,50,51,52] depending on the chemical structure of S-containing organic molecules. For instance, the unbound S3 peak at around 163.1 eV was observed for SAM samples formed from dimethyl disulfides on the Au(111) surface [50]. Moreover, the unbound S3 peak at around 163.5 eV is often observed for unrinsed thiol SAM samples due to the presence of physisorbed molecules on a SAM surface or when a poor solvent was used for thiol adsorption solution [52].

Table 1 summarizes the binding energies of the S1, S2, and S3 peaks and the XPS relative intensities of S 2p/Au 4f and of bound sulfurs (S1 + S2)/Au 4f for 2PymS SAMs on Au(111) formed in different solution pHs. The relative intensity ratios of unbound sulfurs (S3)/Au 4f at solution pH = 2, 7, and 12 were calculated to be 0.00050, 0.00049, and 0.00049, which are nearly the same regardless. Those of bound sulfurs (S1 + S2)/Au 4f were calculated to be 0.00225, 0.00215, and 0.00289, respectively. This means that the surface coverage of 2PymS SAMs chemisorbed on Au(111) formed in a basic solution of pH = 12 significantly increased by 26% and 31% compared with those formed in solution pH = 2 and pH = 7, respectively. Therefore, we concluded that the formation of well-ordered 2PymS SAMs at pH = 12 is mainly driven by a large increase in the chemical interactions between the sulfur headgroup and Au(111) surface and of the van der Waals interactions between molecular backbones (pyrimidine ring), as demonstrated by this STM study (Figure 3).

**Figure 6:**
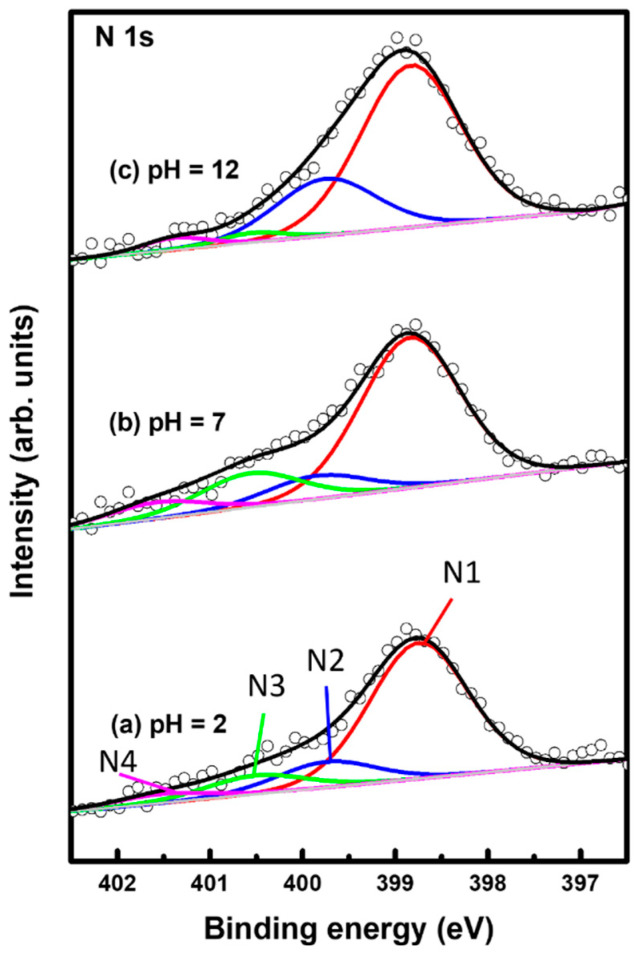
XPS spectra in the region of N 1s of 2PymS SAMs on Au(111) depending on solution pH: (**a**) pH = 2, (**b**) pH = 7, and (**c**) pH = 12. The N 1s spectrum was deconvoluted into four peaks as indicated by N1 (red color), N2 (blue color), N3 (green color), and N4 (pink color).

Figure 6 shows XPS spectra in the region of N 1s of 2PymS SAMs on Au(111) formed for different solution pH values: (a) pH = 2, (b) pH = 7, and (c) pH = 12. The N 1s spectrum was deconvoluted into four peaks as indicated by N1 (red color), N2 (blue color), N3 (green color), and N4 (pink color). Table 2 summarizes the binding energies of N1, N2, N3, and N4 peaks and the XPS relative intensities of (N1 + N2)/Au 4f at different solution pHs. The lowest binding energy peak (N1) showed a strong intensity around 398.8 eV due to the deprotonated N atoms in the pyrimidine ring, whereas the second lowest binding energy peak (N2) at around 399.8 eV is due to the N atoms in the pyrimidine ring interacting with the Au(111) surface, as suggested in the literature [32,54,55]. The side view of the adsorption geometry of 2PymS on Au(111) in Figure 4d clearly shows that two N atoms of the pyrimidine ring in 2PymS SAMs with a upright geometry have a deprotonated form, whereas, in a tilted adsorption geometry, one N atom has a deprotonated form and the other interacts with the Au(111) surface. On the other hand, the higher biding energy peaks (N3 and N4) showing a weak intensity at around 400.5 eV and 401.5 eV are due to the N atoms in different degrees of H acceptance and the protonated N atoms in the pyrimidine ring due to a different adsorption geometry, respectively [55]. The relative intensity of N1/Au 4f for SAMs formed at pH =2, 7, and 12 was 0.00370, 0.00405, and 0.00420, respectively, which increased with solution pH. This result means that the degree of structural order of SAMs significantly increased when increasing the amount of deprotonated N atoms, as revealed by our STM and XPS results. Moreover, the relative intensities of (N1 + N2)/Au 4f for SAMs also increased with increasing solution pH, to 0.00437, 0.00468, and 0.00567, respectively. Interestingly, the relative intensity of (N1 + N2)/Au 4f for highly ordered SAMs (Figure 3c) formed at pH = 12 was 0.00567, which is 29.8% larger than that (0.00437) of poorly ordered SAMs (Figure 3a) formed at pH 2. From our XPS measurements, we clearly demonstrated that the adsorption of DPymDS molecules on Au(111) led to the formation of 2PymS (thiolate) SAMs via the cleavage of the disulfide (S-S) bonds in DPymDS and most N atoms in the pyrimidine rings in 2PymS SAMs existed in the deprotonated form.

## 3. Experimental

### 3.1. Synthesis of DPymDS

DPymDS was synthesized via the oxidation of 2PymSH according to a previously reported method [56], and the chemical structure of DPymDS is shown in Figure 1. 2PymSH (22.9 g, 0.2 mol) was dissolved in ethyl acetate (500 mL) using an ultrasonic sonicator at 40 °C for 30 min. Sodium iodide (3 g) was added, followed by the gradual addition of a 35 wt% hydrogen peroxide solution (0.2 mol) at room temperature (RT). The resulting solution was stirred at RT for 45 min and the loss of starting material, 2PymSH, was confirmed using thin layer chromatography (TLC). Afterward, the solution was extracted twice with ethyl acetate (150 mL each time), and the organic layer was washed with saturated brine solution (100 mL). The separated organic layer was dehydrated with sodium sulfate, and the yellowish solid product was removed via filtration and dried (21.8 g, 98% yield). The product was confirmed using ^1^H-nuclear magnetic resonance (NMR).

### 3.2. Preparation of 2PymS SAMs on Au(111)

Single-crystal Au(111) substrates were obtained via the thermal evaporation of gold on freshly cleaved mica sheets pre-heated to 570 K under a vacuum condition of approximately 10^−5^ Pa [4]. 2PymS SAMs were prepared by immersing clean Au(111) substrates in 0.001, 0.01, and 1 mM ethanolic solutions containing DPymDS at RT for 2 h. To understand the effect of solution pH, SAM samples were also prepared by dipping the Au(111) substrates in a 0.01 mM ethanolic solution at RT for 2 h as a function of solution pH: pH = 2 (acidic), 7 (neutral), and 12 (basic). The pH 2 and pH 12 solutions were adjusted by adding 6 M HCl solution or NaOH powder, respectively. The prepared SAM samples were thoroughly rinsed with ethanol and dried under a high-purity N_2_ gas stream.

### 3.3. STM and XPS Measurements

STM imaging was conducted under ambient conditions at room temperature using a NanoScope E (Veeco, Santa Barbara, CA, USA), equipped with a commercially available Pt/Ir tip. The constant current mode was employed, and bias voltages ranging from 300 to 500 mV were applied, along with tunneling currents ranging from 400 to 600 pA. XPS measurements were performed using the K-alpha plus system (Thermo Scientific, Waltham, MA, USA) with a monochromatic Al K_α_ X-ray source (1486.6 eV). The resulting XPS spectra were calibrated based on the Au 4f_7/2_ peak (84.0 eV).

## 4. Conclusions

The formation and surface structure of 2PymS SAMs on Au(111) via the adsorption of DPymDS molecules depending on solution concentration and pH were investigated using STM and XPS. STM observations revealed that the formation and structural order of 2PymS SAMs were markedly influenced by solution concentration. The SAMs formed in 0.001 mM ethanol mainly contained a disordered phase with many bright islands over the surface, whereas those formed in a 0.01 mM ethanol solution had a relatively uniform and ordered phase. The SAMs formed in a 1 mM ethanol solution were mainly composed of an ordered phase and many bright islands. It was suggested that the solution concentration of a 0.01 mM solution is more suitable for observing the molecular-scale adsorption behaviors of DPymDS molecules on Au(1111) depending on solution pH. The SAMs formed in a 0.01 mM solution at pH 2 were composed of a fully disordered phase with many irregular and bright aggregates, whereas those formed at pH 7 had small ordered domains and many bright islands. As the solution pH increased from pH 7 to pH 12, the surface structures of 2PymS SAMs were remarkably changed from small ordered domains to large ordered domains, which can be assigned to a (4√2 × 3)R51° structure. In addition, XPS measurements clearly demonstrated that 2PymS (thiolate) SAMs were formed via the cleavage of the disulfide (S-S) bond in DPymDS, and the structural order of the SAMs increased with an increase in the amount of deprotonated N atoms in the pyrimidine rings in 2PymS SAMs. Our results obtained here will provide a deep understanding of the molecular self-assembly behaviors and adsorption structures of DPymDS molecules on Au(111).

## Figures and Tables

**Table 1:** XPS peaks in the S 2p region of 2MPM SAMs on Au(111) and XPS relative intensities of S 2p/Au 4f and of bound sulfurs (S1 + S2)/Au 4f at different solution pHs.

pH Condition	S 2p Species	Peak (eV) ^a^	S 2p/Au 4f ^b^	S1 + S2/Au 4f ^b^
pH = 2	S1	161.29	0.00029	0.00225
	S2	162.12	0.00196	
	S3	163.58	0.00050	
pH = 7	S1	161.24	0.00044	0.00215
	S2	162.07	0.00171	
	S3	163.38	0.00049	
pH = 12	S1	160.95	0.00057	0.00289
	S2	162.04	0.00232	
	S3	163.66	0.00049	

^a^ S 2p: S 2p_3/2_ only, ^b^ Au 4f: Au 4f_7/2_.

**Table 2:** XPS peaks in the N 1s region of 2MPM SAMs on Au(111) and XPS relative intensities of N 1s/Au 4f and deprotonated nitrogen (N1 + N2)/Au 4f at different solution pHs.

pH Condition	N 1s Species	Peak (eV)	N 1s/Au 4f ^a^	(N1 + N2)/Au 4f ^a^
pH = 2	N1	398.74	0.00370	0.00437
	N2	399.74	0.00067	
	N3	400.51	000051	
	N4	401.52	0.00024	
pH = 7	N1	398.84	0.00405	0.00468
	N2	399.85	0.00063	
	N3	400.56	0.00091	
	N4	401.57	0.00044	
pH = 12	N1	398.83	0.00420	0.00567
	N2	39975	0.00147	
	N3	400.55	0.00016	
	N4	401.38	0.00020	

^a^ Au 4f: Au 4f_7/2_.

## Data Availability

The data presented in this study are available in this article.
